# Age at First Reproduction Predicts Sex‐Specific Parasite Infection Trajectories Through Adulthood

**DOI:** 10.1002/ece3.74092

**Published:** 2026-08-13

**Authors:** Francisco Ruiz‐Raya, Sarah J. Burthe, Hannah Ravenswater, Fiona Greco, Olivia Hicks, Mark Newell, Carrie Gunn, Francis Daunt, Emma J. A. Cunningham

**Affiliations:** ^1^ Ashworth Laboratories, School of Biological Sciences, Institute of Ecology and Evolution University of Edinburgh Edinburgh UK; ^2^ UK Centre for Ecology and Hydrology Penicuik UK; ^3^ School of Ocean Sciences Bangor University Menai Bridge UK

**Keywords:** European shag (*Gulosus aristotelis*), life‐history trade‐offs, longitudinal study, nematodes, selective disappearance, senescence

## Abstract

Parasites can impose substantial fitness costs on hosts, yet infection intensity varies markedly among individuals and across life stages. Age‐related variation in parasite burden may reflect shifts in behaviour or physiology that alter exposure and susceptibility, or arise from selective disappearance of heavily infected individuals. Distinguishing between these processes is essential for understanding the ecological and evolutionary consequences of parasitism in wild populations. Age at first reproduction (AFR) is a fundamental life‐history trait that shapes lifelong infection dynamics through trade‐offs between reproductive investment, somatic maintenance, immune defence and survival. Because reproductive investment and timing often differ between sexes, age at first reproduction may generate sex‐specific infection trajectories across adulthood, yet whether early‐life reproductive strategies are linked to adult parasite dynamics in a sex‐specific manner remains largely unexplored. Using 10 years of individual‐based monitoring in European shags (*Gulosus aristotelis*), we tested whether age at first reproduction predicts within‐individual changes in gastrointestinal nematode burden across adulthood and whether these trajectories differ between sexes. Parasite burdens were quantified by repeated endoscopy, and a within‐subject centring approach was used to separate within‐ from between‐individual age effects and explicitly account for selective disappearance. Age at first reproduction predicted sex‐specific infection trajectories in male but not female shags. Early‐ and modal‐recruiting males showed within‐individual declines in parasite burden with age, whereas late‐recruiting males exhibited progressive increases. A marginal trend in early‐recruiting males suggests possible selective disappearance in this group. Females showed consistent within‐individual declines regardless of age at first reproduction, with no evidence of selective disappearance. These findings provide the first evidence that age at first reproduction is associated with sex‐specific parasite infection trajectories in a long‐lived bird, revealing how early‐life reproductive strategies are linked to adult host–parasite dynamics. Integrating life‐history variation with longitudinal, within‐individual approaches exposes mechanisms that cross‐sectional analyses cannot detect and highlights the population‐level consequences of sex‐specific infection trajectories.

## Graphical abstract

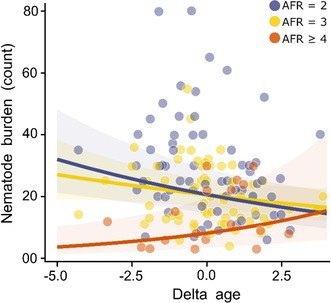

## 1 Introduction

Parasites are ubiquitous in wild animal populations, yet infection intensity varies substantially among individuals and across their lifetimes (Albery et al. 2024; Miller et al. 2007). Age‐related patterns in parasite burden differ widely across taxa, with studies reporting declines, increases, or non‐linear trajectories (e.g., Marzal et al. 2016; Bielański et al. 2017; Freeman‐Gallant and Taff 2017; Ben‐Ami 2019; Hayward et al. 2022). This heterogeneity likely reflects a complex interplay among exposure, susceptibility, and survival, as well as parasite life‐history traits and transmission ecology (Ben‐Ami 2019). Crucially, cross‐sectional analyses can detect age‐specific infection levels, but longitudinal data are essential to distinguish within‐individual changes from population‐level processes such as selective disappearance—the preferential mortality of heavily infected individuals—which can mask true age‐related dynamics (Hayward 2013; Nussey et al. 2008; van De Pol and Verhulst 2006). Identifying the factors shaping individual infection trajectories is therefore central to understanding how parasitism changes across the lifespan and its ecological and evolutionary consequences.

Life‐history traits, particularly early‐life reproductive strategies, may exert lasting influences on infection dynamics and individual health trajectories (Knutie et al. 2017; Leivesley et al. 2019; Lemaître et al. 2015; Lindström 1999; Norris and Evans 2000; Renz and Skevaki 2020; Sheldon and Verhulst 1996). Age at first reproduction (AFR) is a key determinant of lifetime reproductive success and senescence in long‐lived animals (Aubry et al. 2009; Desprez et al. 2014; Kim et al. 2011; Stearns 1992), yet how early‐life reproductive strategies propagate across adulthood to shape adult health trajectories remains an under‐examined question in life‐history ecology. Individuals reproducing early may prioritise reproductive investment at the expense of somatic maintenance and immune function, potentially increasing parasite susceptibility later in life (Knowles et al. 2009; Norris and Evans 2000; Sheldon and Verhulst 1996). Consistent with this, early‐breeding Soay sheep show higher faecal nematode egg counts in later life, suggesting persistent infection costs of early reproduction (Hayward et al. 2010). However, AFR may also reflect intrinsic qualitative differences (sensu Wilson and Nussey 2010), whereby high ‘quality’ individuals (sensu Lailvaux and Kasumovic 2011) reproduce early and maintain effective parasite defences throughout life (Becker and Bradley 2007; Fay et al. 2016). Despite its potential importance, whether AFR predicts individual‐level infection trajectories through adulthood in wild populations remains largely unexplored.

Sex differences in reproductive investment may further modulate the relationship between AFR and infection trajectories. Males and females frequently differ in parasite exposure and susceptibility due to differences in reproductive roles, behaviour, and immune function (Kelehear et al. 2012; Klein 2004; Zuk 2009). Males typically carry higher parasite burdens (e.g., Poulin 1996; Morton and García‐del‐Pino 2013; Rosso et al. 2020), although patterns vary among host and parasite taxa (Butterworth et al. 2024; Kailing et al. 2023; Sanchez et al. 2011; Sheridan et al. 2000). Despite generally lower burdens, females may experience stronger fitness costs from infection (Hicks et al. 2019). These patterns may reflect sex‐specific trade‐offs between reproductive investment and immune function or infection tolerance (Hicks et al. 2019; Mcnamara et al. 2013; Trivers 1972; Zuk and Stoehr 2002). Consequently, early reproduction may generate divergent associations between AFR and adult infection trajectories in males and females.

Here, we tested whether AFR predicts within‐individual infection trajectories across adulthood in the European shag (*Gulosus aristotelis*), a long‐lived seabird with substantial natural variation in AFR, particularly among males (Aebischer 1986). European shags are parasitised by gastrointestinal nematodes acquired through their fish‐based diet (Reed et al. 2008; Burthe et al. 2013), and in our population infection prevalence is near‐universal in both adults and chicks (Burthe et al. 2013; Granroth‐Wilding et al. 2017). Nematode infection reduces daily energy budgets and alters adult foraging behaviour (Hicks et al. 2018; Reed et al. 2008), with males carrying higher burdens but reproductive fitness costs documented only in females (Hicks et al. 2019). Our long‐term, individual‐based dataset, comprising individuals of known age, sex, and reproductive history, provides a rare opportunity to examine whether AFR shapes infection trajectories across adulthood and whether these patterns differ between sexes.

We used a within‐individual centring approach to separate within‐individual age effects from between‐individual differences, quantifying the relative contributions of individual longitudinal trajectories and selective disappearance to population‐level infection patterns (van De Pol and Wright 2009). Based on life‐history theory and the individual quality hypothesis, we generated predictions for how AFR might relate to age‐dependent infection trajectories. If early reproduction imposes lasting costs on somatic maintenance and immune function, early‐recruiting individuals would be expected to show steeper increases in parasite burden with age. Alternatively, if AFR reflects intrinsic individual quality, early‐recruiting individuals (higher quality) may sustain effective parasite defences throughout life, whereas late‐recruiting individuals of more constrained condition may show progressive burden accumulation once the costs of reproduction begin to accrue. Because AFR distributions and life‐history trade‐offs differ markedly between sexes in this species, we expected these patterns to be more pronounced in males, where AFR variation is greater (Aebischer 1986). We specifically tested whether: (*i*) AFR predicts within‐individual changes in nematode burden with age; (*ii*) these associations differ between sexes; and (*iii*) selective disappearance of heavily infected individuals contributes to observed population‐level age patterns.

## 2 Materials and Methods

### 2.1 Study Species and General Procedures

The study was conducted on the Isle of May National Nature Reserve, southeast Scotland (56°11′ N, 2°33′ W), from 2013 to 2022. The European shag (*Gulosus aristotelis*; hereafter shag) is sexually dimorphic, with males 15%–20% larger than females. Shags exhibit bi‐parental care, with both sexes contributing to incubation and chick‐rearing. Since 1997, shag chicks have been ringed with uniquely coded colour rings, generating a population of individually marked adults of known age. Shags typically recruit to the breeding population between ages 2 and 6 years (Aebischer 1986; Potts et al. 1980; Velando and Freire 2002), with an average lifespan of 12–15 years (unpublished data). Peak recruitment occurs at age 3, and no individuals breed before age 2. Age at first reproduction differs between the sexes: among recruiting individuals, approximately 90% of males first breed at age 2, compared with 17% of females (Aebischer 1986; see also Figure S1).

Nest sites were systematically monitored throughout the breeding season (≥ 2 checks per week), recording colour‐ringed adult identities, laying information (clutch initiation and laying dates), and breeding status. Lay and hatching dates were recorded directly during nest checks or estimated by back‐calculation using chick wing length at ringing (typically 20–30 days of age; Daunt 2000). Back‐calculated hatching dates differed from directly observed dates by 1.6 ± 2.4 days (mean ± SD; Grist et al. 2017), which is negligible relative to within‐ and between‐year variation in hatching phenology (Keogan et al. 2021). Annual mean productivity was calculated as the average number of fledglings per incubated nest in unmanipulated monitoring plots independent of study individuals (see Newell et al. 2015 for additional details).

In our study population, natal dispersal is low [90.4% of individuals bred on the Isle of May and a further 6.1% bred ≤ 50 km away (Barlow et al. 2013)]. Intensive monitoring ensures first‐time breeders are unlikely to be missed, and both breeding and non‐breeding individuals are recorded during systematic colony surveys, as colour‐ringed birds of all ages return to the colony regardless of reproductive status, providing strong confidence that assigned AFR values reflect true first breeding attempts. We note, however, that some early breeders that failed before detection may have been missed, though we expect these cases to be few and unlikely to substantially affect results. Adult sexing was conducted by molecular techniques and/or sex‐specific vocalisations observed during nest monitoring, validated by long‐term observer experience and repeated identification across years. All individuals included in this study were ringed as chicks and therefore of known age.

### 2.2 Quantification of Parasite Intensity

Parasite assessments were carried out during breeding seasons from 2013 to 2022 as part of a long‐term individual‐based study (Burthe et al. 2013; Granroth‐Wilding et al. 2015, 2017; Hicks et al. 2019; Acker et al. 2021). Shags are parasitised by gastrointestinal anisakid nematodes, ingested as larvae via their fish diet (Reed et al. 2008; Burthe et al. 2013). Exposure to nematodes begins during the chick stage, when individuals are first fed by their parents, and infection has been documented in birds of all ages (Granroth‐Wilding et al. 2014, 2017). Nematode burden (number of nematodes present in each bird) was quantified using endoscopy on live individuals, performed under licence (Burthe et al. 2013; Granroth‐Wilding et al. 2017; Ravenswater et al. 2025). Mature worms have been identified as *Contracaecum rudolphii*, while larvae are potentially both *C. rudolphii* and *Anisakis simplex* (Reed et al. 2008). Shags return to the same breeding areas annually (Barlow et al. 2013), enabling individual resampling across lifetimes. We analysed 300 endoscopies from 106 known‐age adult shags (55 females, 51 males) between 2013 and 2022. Most birds were sampled in multiple years (62%), resulting in an average of 2.8 endoscopic observations per individual (range: 1–9; see Figure S2 for distribution of sampling effort across individuals and age classes). Individuals were never sampled more than once per year, so observation number equates to number of years sampled.

Endoscopy procedures were performed by a trained operator authorised under the UK Home Office's Animals (Scientific Procedures) Act 1986, typically during late chick rearing. Endoscopy is a reliable method for directly and repeatedly measuring nematode loads in live shags (Burthe et al. 2013), providing accurate worm quantification compared to methods such as faecal egg count (FEC), which can be influenced by worm density and shedding variation (Ghosh et al. 2018). In shags, FEC‐based prevalence and burden estimates are notably lower than endoscopic estimates, with some individuals showing no FEC despite endoscopic evidence of infection (Granroth‐Wilding et al. 2017). Endoscopic methods therefore provide a consistent index for longitudinal comparisons across individuals and years, even if the method does not capture total parasite load, as it consistently quantifies relative burden across individuals and age classes. Previous research in 2011–2012 involved anti‐helminthic treatment of select individuals (Burthe et al. 2013; Granroth‐Wilding et al. 2015); consequently, treated birds were excluded from this study. Control individuals receiving saline were included, as previous studies found no differences between untreated and saline‐treated controls (Granroth‐Wilding et al. 2015). No differences in behavioural or fitness‐related traits have been detected between endoscoped birds and the general population (Burthe et al. 2013).

### 2.3 Statistical Analyses

All analyses were performed in R version 4.2.1 (R Core Team 2022). We used generalised linear mixed models (GLMMs) with Poisson error structure fitted in *glmmTMB* (Brooks et al. 2017), with nematode burden (count data from endoscopic assessments) as the response variable.

To separate within‐ and between‐individual components of age, we applied a within‐individual centring approach (van De Pol and Wright 2009). Age at sampling was decomposed into ∆ age (within‐individual deviation from each individual's mean age at sampling) and average age (capturing between‐individual differences in mean age), both included as continuous predictors in all models. Average annual productivity and within‐year centred laying date were also included as covariates to account for variation in environmental conditions affecting host condition and infection risk (Granroth‐Wilding et al. 2014; Keogan et al. 2021). Random intercepts for individual ID and year accounted for repeated measurements and interannual variation in all models.

Models were fitted within a hypothesis‐driven framework in which each model addressed a pre‐specified biological question. The primary hypothesis concerned whether age at first reproduction (AFR) modifies within‐individual age‐dependent infection trajectories and whether this effect differs between sexes. Baseline age‐dependent infection patterns were first characterised using a model including sex, ∆ age, average age, and their interactions, alongside annual productivity and within‐year centred laying date. AFR was then introduced as a main effect to test whether recruitment timing predicts overall nematode burden independently of age trajectories, and whether its effects operate through baseline differences or slope modification. AFR was modelled as a three‐level factor reflecting biologically interpretable recruitment strategies (Kappes et al. 2021; Nussey et al. 2006): age 2 (early recruits; 82 observations from 31 individuals), age 3 (modal recruits; reference category; 154 observations from 53 individuals), and age ≥ 4 (late recruits; 59 observations from 22 individuals). This captures biologically meaningful differences in recruitment timing while balancing sample sizes within groups. To assess sensitivity to this AFR categorisation, AFR was also fitted as a continuous predictor. This yielded consistent estimates of the AFR × age interaction (Table S1), suggesting that the observed patterns are not driven by discretisation of AFR.

Non‐linear age‐related infection trajectories are biologically plausible: nematode burdens might be expected to decline into prime age as individuals acquire immunity and improve foraging competence, before increasing in old age as immunosenescence occurs (Duerr et al. 2003; Hayward et al. 2022). We therefore evaluated whether quadratic age terms improved model fit. Inclusion of quadratic terms did not improve model fit (linear: AIC = 2425.48; quadratic: AIC = 2430.65; ΔAIC = 5.17), and visual inspection of within‐individual trajectories provided no indication of systematic curvature. Linear age effects were therefore retained throughout.

Sex‐specific models were fitted a priori because AFR distributions differ markedly between sexes and because sex differences in life‐history strategy and nematode burden imply distinct underlying biological processes. Males predominantly recruit at age 2, whereas females show later and less variable recruitment (Aebischer 1986), and the sexes differ in baseline nematode burden (Hicks et al. 2019). Modelling sexes jointly would therefore conflate structurally different AFR distributions and obscure sex‐specific mechanisms. Sex‐specific models retained the same structure as the pooled model, including ∆ age, average age, AFR, their interactions, and environmental covariates as fixed effects, and individual ID and year as random intercepts.

To evaluate whether selective disappearance contributed to cross‐sectional infection patterns, we reparameterised models to include chronological age alongside average age. In this formulation, chronological age represents within‐individual change, while a significant average‐age effect indicates divergence between within‐ and between‐individual slopes, consistent with selective disappearance (van De Pol and Wright 2009; see Supplementary Methods). Model assumptions were assessed using *DHARMa* diagnostics (Hartig 2025). Multicollinearity was assessed using variance inflation factors (VIF range: 1.06–2.72; *performance* package; Lüdecke et al. 2021), indicating no evidence of problematic collinearity. Uncertainty in fixed effects was quantified using parametric bootstrapping (1000 iterations), yielding stable coefficient estimates and confidence intervals. Interaction terms were removed when non‐significant following standard model simplification procedures (Engqvist 2005). Where interactions were supported, group‐specific slopes were estimated using *emmeans* (Lenth 2023) and expressed as incidence rate ratios (IRRs) for interpretability.

Finally, the robustness of the key AFR × ∆ age interaction was specifically evaluated in males, where the late‐recruit (AFR ≥ 4) group is smallest and potentially most sensitive to sample structure. First, a leave‐one‐individual‐out jackknife procedure was applied, refitting the model after sequential removal of each individual and re‐estimating the interaction term, providing a direct test of whether inference is driven by any single individual. Second, a permutation test was conducted to evaluate whether the observed interaction could arise by chance under random assignment of AFR. AFR categories were permuted at the individual level, preserving within‐individual observation structure, and the model was refitted across 1000 iterations to generate a null distribution of the interaction coefficient (Figure S3). The proportion of permuted coefficients equal to or exceeding the observed value constituted a one‐tailed permutation *p*‐value, with a one‐tailed test used because the direction of the interaction was specified a priori.

## 3 Results

We used 144 observations from 51 males and 151 observations from 55 females. Mean nematode burden across all observations was 20.1 ± 0.8 (mean ± SE; range: 1–80), with males exhibiting higher burdens (23.7 ± 1.2) than females overall (16.7 ± 0.9). AFR distributions differed between sexes, with early recruitment (AFR = 2) more common in males than females (see Figure S1).

### 3.1 Age Structure of Nematode Burden

Nematode burden declined significantly with both within‐individual age (∆ age: β = −0.10, 95% CI = −0.16 to −0.04) and between‐individual age (average age: β = −0.15, 95% CI = −0.28 to −0.03; Table S2). Males had higher nematode burdens than females overall (β = 0.34, 95% CI = 0.15–0.51), but there was no evidence that overall age trajectories differed between sexes, as neither sex × ∆‐age nor sex × average‐age interactions were supported (Table S2). Overall, early recruits (AFR 2) carried higher nematode burdens than the modal recruitment group (β = 0.25, 95% CI = 0.04–0.47), whereas late recruits (AFR ≥ 4) did not differ from the modal group (β = 0.08, 95% CI = −0.20 to 0.34; Table S3). Our analyses concerning whether AFR modified age‐dependent infection trajectories are addressed in Sections 3.2 and 3.3.

### 3.2 Age at First Reproduction Predicts Male Infection Trajectories

**FIGURE 1:**
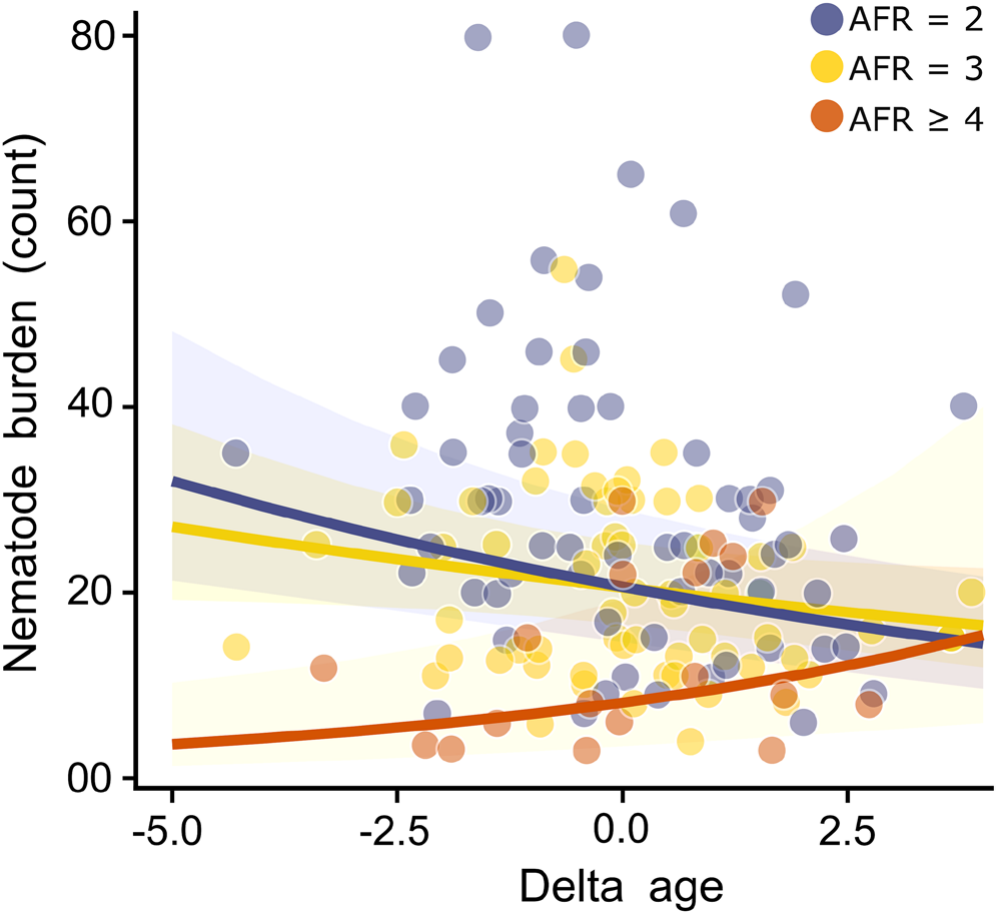
Trajectories of nematode infection in adult male European shags. Nematode burden trajectories varied with age at first reproduction (AFR) in males. Points represent raw data for individual nematode burdens, slightly jittered to improve visibility. Thick solid lines show the predicted changes in nematode burden with age for each AFR group. Shaded areas denote 95% confidence intervals around predicted values.

We found that age‐related infection trajectories differed among recruitment strategies in males. Specifically, late‐recruiting males (AFR ≥ 4) exhibited significantly more positive within‐individual age trajectories than the modal recruitment group (∆ age × AFR ≥ 4: β = 0.37; 95% CI = 0.21–0.55; Figure 1), whereas early‐recruiting males (AFR 2) did not differ from the modal group (∆ age × AFR 2: β = −0.05; 95% CI = −0.14 to 0.04; Figure 1; see Figure S4 for chronological age scale). More specifically, early‐recruiting males showed a significant annual decline in nematode burden (β = −0.15; 95% CI = −0.24 to −0.07; 14% decrease per year, IRR = 0.86, 95% CI = 0.79–0.94). The modal group (AFR 3) showed a marginally significant decline (β = −0.10; 95% CI = −0.19 to 0.00; 9% decrease per year, IRR = 0.91, 95% CI = 0.82–1.00). In contrast, late‐recruiting males exhibited a significant within‐individual increase (β = 0.28; 95% CI = 0.09–0.47; 32% increase per year, IRR = 1.32, 95% CI = 1.09–1.59).

The AFR × within‐individual age interaction in males was robust to removal of individual birds, with jackknife estimates remaining consistently positive across all iterations (95% range: 0.34–0.39), indicating that the effect was not driven by any single influential individual. A permutation test further confirmed this result: the observed interaction coefficient (β = 0.37) fell in the extreme tail of the null distribution generated by randomly reassigning AFR labels among individuals (*p* = 0.026; Figure S3).

There was also evidence that between‐individual age effects varied among recruitment groups. Early‐recruiting males showed a negative average‐age interaction relative to the modal group (β = −0.49, 95% CI = −0.89 to −0.07), meaning that, across individuals, those with a higher mean sampling age tended to carry lower nematode burdens. No comparable cross‐sectional age pattern was detected in the modal or late‐recruiting groups (Table S4).

### 3.3 Female Infection Dynamics Are Independent of AFR

**FIGURE 2:**
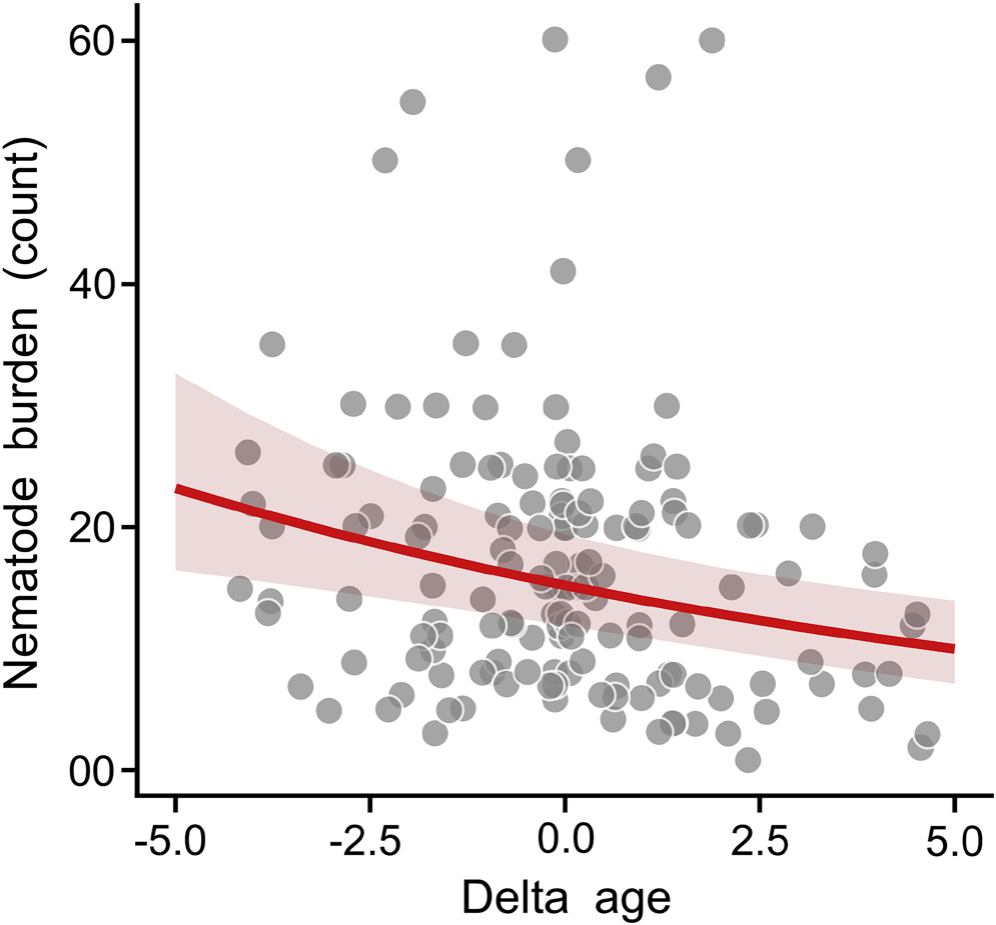
Trajectories of nematode infection in adult female European shags. Nematode burden consistently declined with age across age at first reproduction (AFR) groups in females. Points represent raw data for individual nematode burdens, slightly jittered to improve visibility. Thick solid line shows the predicted changes in nematode burden with age. Shaded areas denote 95% confidence intervals around predicted values.

Females showed a consistent within‐individual decline in nematode burden with age (∆ age: β = −0.15, 95% CI = −0.23 to −0.06), with a 14% decrease per year (IRR = 0.86, 95% CI = 0.80–0.94; Figure 2; see Figure S5 for chronological age scale). Between‐individual effects were similarly negative (average age: β = −0.21, 95% CI = −0.38 to −0.05), indicating lower nematode burdens among older females. Neither AFR × ∆ age nor AFR × average age interactions were supported in females (confidence intervals overlapped zero in all interaction terms; Table S5), confirming that infection trajectories were independent of recruitment strategies. Neither early nor late female recruits differed significantly from the modal recruitment group in overall nematode burden (Table S5).

### 3.4 Evidence for Selective Disappearance

To assess whether cross‐sectional age patterns reflected selective disappearance rather than within‐individual change, we fitted alternative models including chronological age and average age simultaneously. In females, the average‐age term was not significant in this model (β = 0.03; 95% CI = −0.19 to 0.26; see Chronological‐age model in Table S5), indicating no detectable divergence between cross‐sectional and within‐individual age effects. Similarly, in males, there was little evidence that selective disappearance contributed substantially to observed age‐related infection patterns. The main effect of average age was not significant (β = 0.17; 95% CI = −0.07 to 0.41), and interactions between AFR and average age were weak, with only a marginal trend among early‐recruiting males (β = −0.38, 95% CI = −0.82 to 0.03; Table S4).

## 4 Discussion

Understanding how parasite burdens change across individual lifespans is fundamental to predicting host–parasite dynamics and their fitness consequences in wild populations (Duerr et al. 2003; Penczykowski et al. 2016). Using 10 years of individual‐based monitoring in European shags, we show that age at first reproduction (AFR) shapes sex‐specific nematode infection trajectories throughout adulthood, in ways that differ strikingly between the sexes. In males, within‐individual trajectories diverged by AFR: both modal‐recruiting (AFR = 3) and early‐recruiting males showed declining nematode burdens with age, whereas late‐recruiting males exhibited progressive increases. Females, by contrast, showed a consistent age‐related decline regardless of recruitment age. Crucially, selective disappearance played no detectable role overall, indicating that these patterns are shaped mainly by within‐individual changes with age, although a marginal trend in early‐recruiting males suggests a survival cost of high infection in this group that needs further confirmation. Together, these findings are consistent with several non‐exclusive mechanisms, including pre‐existing individual quality differences, shifts in investment trade‐offs over reproductive lifespan, or early‐life conditions that independently shape both recruitment timing and adult infection dynamics.

### 4.1 Age at First Reproduction and Male Infection Trajectories

The divergence of within‐individual infection trajectories by AFR in males is one of the most striking findings of our study, and points to a link between reproductive life‐history timing and infection dynamics. Several non‐exclusive mechanisms may account for this pattern, including differences in physiological condition or exposure, and shifts in investment trade‐offs across the reproductive lifespan. Individuals in better condition or with more favourable early‐life histories may recruit early while sustaining robust parasite defences throughout life. We found that both early‐ and modal‐recruiting males showed significant within‐individual declines in nematode burden with age (14% and 9% per year, respectively) and these trajectories did not differ significantly from each other, suggesting that early recruitment is not associated with a detectably different infection trajectory from the modal pattern. This declining trend in early‐recruiting males is consistent with sustained investment throughout life and may reflect a balance between ongoing parasite defence and the physiological demands of early breeding. Late‐recruiting males, by contrast, showed an increase in burden with age, a pattern consistent with accelerated immune senescence or growing infection susceptibility once breeding begins in individuals of more constrained condition.

AFR is frequently associated with individual quality in long‐lived seabirds, with lower‐quality individuals delaying recruitment and performing more poorly in later life (Becker and Bradley 2007; Limmer et al. 2010; Aubry et al. 2011; Fay et al. 2016), which is consistent with state‐dependent variation in performance with age in shags (Daunt et al. 1999). The burden increases found in late‐recruiting males may therefore reflect cumulative costs of reproductive investment in individuals least able to buffer them, potentially exacerbated by reduced foraging efficiency, limited territory quality, or constrained immune investment (Forbes et al. 2016).

Differential parasite exposure may further contribute to these trajectories. Individuals recruiting at older ages might experience different exposure regimes prior to first breeding, for instance, through habitat use or foraging strategies that limit parasite acquisition and encounter elevated exposure only once reproduction begins. Age‐related improvements in foraging efficiency (Daunt et al. 2007) might reduce parasite exposure across all groups as individuals accumulate experience, yet these gains appear insufficient to prevent burden accumulation in late‐recruiting males, suggesting that condition‐dependent or reproductive cost processes override any exposure‐reduction benefit in this group. These exposure‐based mechanisms are not independent of individual quality: access to lower‐risk foraging habitat and more efficient prey acquisition are themselves likely to reflect individual condition and competitive ability, meaning that quality‐based and exposure‐based pathways may reinforce one another rather than operate in isolation. Disentangling these pathways is not straightforward, however, since parasite burdens accumulated early in life could themselves influence recruitment timing, a possibility that highlights the value of longitudinal data spanning the pre‐recruitment period, or of experimental manipulation of early‐life conditions. Recent work in this population has shown that early‐life conditions predict adult parasite burdens in males but not females (Ravenswater et al. 2025), suggesting that early‐life processes and adult life‐history strategies jointly shape male infection trajectories, with recruitment timing potentially amplifying effects established before first breeding.

We found no strong evidence for selective disappearance overall, but a marginal trend in early‐recruiting males warrants consideration. In females, the absence of any divergence between within‐ and between‐individual age effects confirms that the consistent burden decline reflects genuine within‐individual improvement. The directional trend in early‐recruiting males is consistent with heavily infected individuals in this group being less likely to persist, although the confidence interval includes zero and confirmation may require larger samples. If confirmed, this would suggest that recruiting young imposes compounding physiological demands (Knowles et al. 2009; Norris and Evans 2000; Sheldon and Verhulst 1996), and that nematode parasitism may mediate survival trade‐offs associated with early breeding documented in other long‐lived species (Blomquist 2009; Ryan et al. 2024). We interpret this tentatively, however, as direct tests of survival would be needed to evaluate it properly. In contrast, the weak evidence for selective disappearance in late‐recruiting males and females confirms that the burden increases in late‐recruiting males and the consistent female decline reflects within‐individual trajectories rather than compositional artefacts.

### 4.2 Age‐Related Decline in Female Parasite Burden

In contrast to the complex, AFR‐dependent picture in males, female shags showed a consistent pattern: parasite burden declined with age within individuals, and this trajectory was entirely independent of recruitment age. Given near‐universal early‐life exposure to nematodes in our population (98% prevalence in nestlings; Granroth‐Wilding et al. 2017), recurrent infection throughout life is highly likely, and the within‐individual decline in nematode infection is most parsimoniously explained by acquired immunity (i.e., the accumulation of parasite‐specific immune responses that reduce susceptibility over time; Anderson and May 1985; Lee 2006; Lee et al. 2008; Woolhouse 1992). This interpretation is supported by evidence of antibody maintenance with age in other seabird systems (Staszewski et al. 2007; Ramos et al. 2014). These processes presumably operate in males too, yet in males they may be modulated by stronger state‐dependent effects linked to reproductive timing, underlining why a sex‐specific, longitudinal framework is essential for interpreting population‐level infection patterns.

Senescence patterns differ between male and female seabirds, and recruitment‐age effects on longevity have been found to vary by sex (Kim et al. 2011), with immunity–reproduction trade‐offs apparently stronger in males (Mcnamara et al. 2013). In European shags, age at first reproduction also differs markedly between the sexes: among recruiting individuals, approximately 90% of males first breed at age 2, compared with only 17% of females (Aebischer 1986). Such sex differences in the timing of reproduction may contribute to the divergent infection trajectories observed in our study. The broader implication is that life‐history heterogeneity among males creates a wider range of trajectories than in females, with consequences that ripple through into population‐level infection dynamics. These sex‐specific adult patterns align with early‐life influences reported in the same population, where early‐life conditions have been found to predict adult parasite burdens in males but not females (Ravenswater et al. 2025).

Foraging ecology can represent an additional axis of sex difference in parasite exposure. Female shags forage further from the colony and make longer trips than males (Carravieri et al. 2020; Soanes et al. 2014), likely encountering prey assemblages with distinct parasite loads, since nematode transmission depends directly on which fish species and size classes are consumed (Leung and Koprivnikar 2016; Vitone et al. 2004). Importantly, age‐related shifts in diet composition and foraging habitat use and efficiency (Daunt et al. 2007) could incidentally reduce parasite intake as individuals accumulate experience (Albery et al. 2024). Early‐recruiting males might be particularly exposed in this respect, beginning to breed before reaching peak foraging competence and potentially being constrained to higher‐risk foraging areas or prey (Forbes et al. 2016). Thus, sex‐specific foraging behaviour might independently modulate the parasite exposure trajectories underlying the patterns described above.

### 4.3 Ecological and Population‐Level Implications

Our results confirm that within‐individual longitudinal approaches can resolve mechanisms that cross‐sectional studies cannot detect. Within‐individual trajectories in individual traits are frequently masked by selective disappearance when studied cross‐sectionally across mammal and bird taxa (Bouwhuis et al. 2009; Nussey et al. 2008; Nussey et al. 2009), and parasite burden is no exception. Without longitudinal partitioning, the divergent infection trajectories of male AFR groups in our study population, or the within‐individual nature of female decline, would be invisible or misattributed to population‐level compositional change (van De Pol and Verhulst 2006). The limited role of selective disappearance in our study suggests that survival consequences of nematode burden in this population are more subtle or condition‐dependent than acute costs, or that costs accumulate over timescales exceeding our 10‐year window, a possibility that survival models could directly evaluate. The consistent within‐individual decline in female burden implies that females carry diminishing infections throughout life, likely buffered by acquired immunity. In males, the divergent trajectories by AFR reveal a population structured by qualitatively different infection pathways, an individual heterogeneity that may influence how different life‐history strategies respond to environmental perturbations affecting prey availability, breeding phenology, or immune investment.

These results contribute to a broader question in life‐history ecology: how does early reproductive timing shape long‐term health trajectories across wild vertebrates? The individual quality hypothesis predicts that high‐quality individuals would be able to recruit early (Becker and Bradley 2007; Fay et al. 2016) and maintain effective physiological defences throughout life, and our finding that late‐recruiting rather than early‐recruiting males show progressive burden accumulation is consistent with this framework extending to parasite dynamics. Similarly, early‐recruiting males in our study show declining trajectories comparable to the modal group rather than the burden increases that the reproductive cost hypothesis would predict, reinforcing the interpretation that AFR in this system primarily reflects individual quality and provides less support for a simple reproductive cost explanation. This pattern contrasts with evidence from Soay sheep, where early‐breeding females carry higher nematode burdens (faecal counts) in later life (Hayward et al. 2010), a pattern consistent with the reproductive cost framework, suggesting that the relative importance of individual quality versus reproductive costs in shaping infection trajectories may vary across taxa and life‐history contexts. More broadly, integrating developmental and adult processes, including how early‐life conditions set infection trajectories subsequently modified by reproductive decisions and immune investment, will be essential for understanding how parasitism shapes fitness across the lifespan in wild populations.

## 5 Conclusion

This study provides longitudinal evidence that age at first reproduction shapes sex‐specific parasite infection trajectories in a wild seabird. Late‐recruiting males experienced progressively increasing nematode burdens with age, while early‐recruiting males showed within‐individual declines comparable to the modal group; females' parasite burden declined consistently with age regardless of recruitment timing. By partitioning within‐ from between‐individual effects and finding no overall evidence of selective disappearance in either sex, we show that these trajectories may reflect genuine biological change occurring within individuals across their lifespans. These results highlight how reproductive timing and sex are associated with parasite dynamics of long‐lived animals, and confirm that longitudinal, within‐individual approaches are essential for revealing the mechanisms that cross‐sectional studies obscure.

## Author Contributions

**Francisco Ruiz‐Raya:** conceptualisation (lead), data curation (lead), formal analysis (lead), investigation (supporting), methodology (supporting), validation (lead), visualisation (lead), writing – original draft (lead), writing – review and editing (lead). **Sarah J. Burthe:** data curation (equal), funding acquisition (equal), project administration (equal), resources (equal), supervision (equal), writing – review and editing (equal). **Hannah Ravenswater:** investigation (equal), methodology (equal), writing – review and editing (equal). **Fiona Greco:** investigation (equal), methodology (equal), writing – review and editing (equal). **Olivia Hicks:** investigation (equal), methodology (equal), writing – review and editing (equal). **Mark Newell:** investigation (equal), methodology (equal), writing – review and editing (equal). **Carrie Gunn:** investigation (equal), methodology (equal), writing – review and editing (equal). **Francis Daunt:** data curation (equal), funding acquisition (equal), project administration (equal), resources (equal), supervision (equal), writing – review and editing (equal). **Emma J. A. Cunningham:** data curation (equal), funding acquisition (lead), project administration (lead), resources (equal), supervision (equal), writing – review and editing (equal).

## Funding

This work was supported by post‐doctoral funding from Natural Environment Research Council grant number NE/V001779/1.

## Conflicts of Interest

The authors declare no conflicts of interest.

## Supporting information


**Figure S1:** Histogram showing variation in the age at first reproduction (AFR) in our study population for all individuals (females and males together; A, B), female shags (C, D) and male shags (E, F).
**Figure S2:** (A) Heat map showing the age ranges at which individuals were sampled. Each row represents an individual bird sampled at different ages. (B) Histogram showing the number of individuals that were sampled at each age. (C) Histogram showing the number of individuals in each average age class. Dotted red lines denote mean values.
**Figure S3:** Permutation test of the interaction between age at first reproduction (AFR) and within‐individual age on nematode burden in males. The histogram shows the null distribution of interaction coefficients obtained by randomly permuting AFR labels among individuals (*n* = 998 permutations), while preserving within‐individual observation structure. The observed coefficient (solid red line; β = 0.37) lies at the upper tail of the distribution, exceeding 95% of permuted values (*p* = 0.026). The dashed line indicates the 95th percentile of the null distribution.
**Figure S4:** Effects of age on within‐individual changes in nematode burden in male shags (shown on chronological age scale based on the Chronological‐age model; Table S3). Points represent raw data for individual parasite burdens, slightly jittered to improve visibility. Thick solid lines show the predicted changes with age at different AFR. Shaded areas denote 95% confidence intervals around predicted values.
**Figure S5:** Effects of age on within‐individual changes in nematode burden in female shags (shown on chronological age scale based on the Chronological‐age model; Table S2). Points represent raw data for individual parasite burdens, slightly jittered to improve visibility. Shaded area denotes 95% confidence intervals around predicted values.
**Table S1:** Sensitivity analysis in which age at first reproduction (AFR) was modelled as a continuous variable rather than as the categorical variable used in the main analyses. Separate GLMMs were fitted for males and females to test the effects of AFR and age on nematode burdens in European shags. Coefficients, 95% confidence intervals (CIs), and *p*‐values were estimated using parametric bootstrapping (1000 iterations). Significant effects are highlighted in bold.
**Table S2:** Baseline GLMM testing effects of age structure (delta age, average age), sex, productivity, and laying date on nematode burden. Sex interactions with age terms were included to test for sex‐specific age trajectories.
**Table S3:** Extended GLMM including age at first reproduction (AFR). Model extending the baseline GLMM by including age at first reproduction (AFR; categorical: age 2, age 3 [reference], and age ≥ 4) as a fixed effect, along with interactions between AFR and age structure terms to test whether recruitment timing modifies age‐dependent infection trajectories. All other fixed and random effects are identical to the baseline model.
**Table S4:** Summary of GLMMs testing the effects of age at first reproduction (AFR) and age on nematode burdens in male European shags. Coefficients, 95% confidence intervals (CIs) and *p*‐values were calculated by parametric bootstrapping (1000 iterations). Significant effects are highlighted in bold.
**Table S5:** Summary of GLMMs testing the effects of age at first reproduction (AFR) and age on nematode burdens in female European shags. Interactions between AFR and delta‐age, and between AFR and average‐age, were dropped from models when non‐significant (see Methods). Coefficients, 95% confidence intervals (CIs) and *p*‐values were calculated by parametric bootstrapping (1000 iterations). Significant effects are highlighted in bold.

## Data Availability

The datasets generated and analysed during the current study are available online at https://doi.org/10.6084/m9.figshare.32982668.
