# Supplementary material for: Age at First Reproduction Predicts Sex‐Specific Parasite Infection Trajectories Through Adulthood
Source: Ecol Evol. 2026 Aug 13;16(8):e74092. doi: 10.1002/ece3.74092 (PMC13472769; doi:10.1002/ece3.74092)
Supplement: Supplementary file 1 [file ECE3-16-e74092-s001.docx]

Supplementary Materials for:

**Age at first reproduction predicts sex-specific parasite infection trajectories through adulthood**

Francisco Ruiz-Raya*^1,2^, Sarah J. Burthe^3^, Hannah Ravenswater^1^, Fiona Greco^1^, Olivia Hicks^4^, Mark Newell^3^, Carrie Gunn^3^, Francis Daunt^3^ and [Emma J. A. Cunningham](https://www.nature.com/articles/s41598-025-85152-6#auth-Emma_J__A_-Cunningham-Aff1)^1^.

^1^ *Institute of Ecology and Evolution; School of Biological Sciences, University of Edinburgh, Ashworth Laboratories, King’s Buildings, Charlotte Auerbach Road, Edinburgh, EH9 3FL, UK*

*2 Present address: School of Biodiversity, One Health and Veterinary Medicine; Graham Kerr Building, University of Glasgow, Glasgow G12 8QQ, UK*

*3 UK Centre for Ecology & Hydrology, Bush Estate, Penicuik, EH26 0QB, UK*

*4* *School of Ocean Sciences, Bangor University, Menai Bridge LL59 5AB, UK*

***Corresponding author:** Francisco.ruiz-raya@glasgow.ac.uk

A. **SUPPLEMETARY METHODS**

**A1. Distinguishing within- and between-individual age effects**

Our primary objective was to disentangle how an individual’s age of first reproduction is associated with parasite burden across adulthood (i.e., infection trajectories; within-individual aging effects) from differences in burden between individuals of varying ages (cross-sectional comparison; between-individual age differences). To achieve this, we used within-individual centering, a well-validated approach for longitudinal data (van De Pol and Verhulst 2006; van De Pol and Wright 2009a).

In traditional linear mixed-effects models (LMMs), the fixed-effect coefficient for a time-varying predictor (e.g., age) conflates two distinct processes: (1) Within-individual change: How a trait (e.g., parasite burden) changes as an individual ages; (2) Between-individual differences: How older individuals systematically differ from younger ones. This can be described by the following regression model:

$$y_{ij}=\beta_{0}+\beta_{1}x_{ij}+u_{0j}+\varepsilon_{ij}$$

Where: $\beta_{0}$ is the fixed intercept, $\beta_{1}$ is the fixed effect of predictor $x$ (e.g., age), $u_{0j}$ is the random intercept for individual $j$, $\varepsilon_{ij}$ is the residual error for observation $i$ of individual $j$.

To isolate these components, we decomposed chronological age ($x_{ijk}$) into:

a. **Average age** ($\bar{x}_{k}$): The mean age of individual *k* across all observations (between-individual component).
b. **Delta age** ($x_{ijk}-\bar{x}_{k}$): The deviation of each observation from the individual’s mean age (within-individual component).

This yielded the following model:

$$y_{ijk}=\beta_{0}+\beta^{w}(x_{ijk}-\bar{x}_{k})+\beta^{b}\bar{x}_{k}+u_{0k}+e_{ijk}$$

where:

$\beta^{w}$: The within-individual effect of age (e.g., how parasite burden changes as the same individual ages).

$\beta^{b}$: The between-individual effect (e.g., differences in burden between individuals with older vs. younger mean ages).

$u_{0k}$: Random intercepts accounting for individual-specific baseline burden.

$e_{ijk}$: Residual error.

This decomposition allows us to interpret $\beta^{w}$ as the effect of aging within individuals, and $\beta^{b}$as how individuals with higher average age differ from those with lower average age, which may also reflect non-random survival (e.g., selective disappearance; see below).

Testing for Selective Disappearance:

To evaluate whether age-related patterns in parasite burden could arise from selective mortality (e.g., highly infected individuals dying younger), we reformulated the model to explicitly compare $\beta^{w}$ and $\beta^{b}$:

$$y_{ijk}=\beta_{0}+\beta^{w}x_{ijk}+(\beta^{b}-\beta^{w})\bar{x}_{k}+u_{0k}+e_{ijk}$$

Here, the coefficient for $\bar{x}_{k}(\beta^{b}-\beta^{w})$ tests whether within- and between-individual effects diverge. A significantly coefficient for this term would suggest that individuals with certain values of the outcome variable (e.g., parasite burden) are more likely to disappear from the sample, implying selective mortality may be influencing age-related patterns.

B. **SUPPLEMETARY FIGURES**

**Figure S1.** Histogram showing variation in the age at first reproduction (AFR) in our study population for all individuals (females and males together; A, B), female shags (C, D) and male shags (E, F).

**A**

**B**

**C**

**Figure S2.** (A) Heat map showing the age ranges at which individuals were sampled. Each row represents an individual bird sampled at different ages. (B) Histogram showing the number of individuals that were sampled at each age. (C) Histogram showing the number of individuals in each average age class. Dotted red lines denote mean values.

**Figure S3**. Permutation test of the interaction between age at first reproduction (AFR) and within-individual age on nematode burden in males. The histogram shows the null distribution of interaction coefficients obtained by randomly permuting AFR labels among individuals (n = 998 permutations), while preserving within-individual observation structure. The observed coefficient (solid red line; β = 0.37) lies at the upper tail of the distribution, exceeding 95% of permuted values (p = 0.026). The dashed line indicates the 95th percentile of the null distribution.

**Figure S4.** Effects of age on within-individual changes in nematode burden in male shags (shown on chronological age scale based on the Chronological-age model; Table S3). Points represent raw data for individual parasite burdens, slightly jittered to improve visibility. Thick solid lines show the predicted changes with age at different AFR. Shaded areas denote 95% confidence intervals around predicted values.

**Figure S5.** Effects of age on within-individual changes in nematode burden in female shags (shown on chronological age scale based on the Chronological-age model; Table S2). Points represent raw data for individual parasite burdens, slightly jittered to improve visibility. Shaded area denotes 95% confidence intervals around predicted values.

C. **SUPPLEMETARY TABLES**

**Table S1.** Sensitivity analysis in which age at first reproduction (AFR) was modelled as a continuous variable rather than as the categorical variable used in the main analyses. Separate GLMMs were fitted for males and females to test the effects of AFR and age on nematode burdens in European shags. Coefficients, 95% confidence intervals (CIs), and *p*-values were estimated using parametric bootstrapping (1000 iterations). Significant effects are highlighted in bold.

|  | Males | | | Females | | |
| --- | --- | --- | --- | --- | --- | --- |
| Parameter | *β* | 95% CI | *p*-value | *β* | 95% CI | *p*-value |
| *Intercept* | **2.96** | **[2.76, 3.16]** | **<0.001** | **2.72** | **[2.50, 2.95]** | **<0.001** |
| AFR (continuous) | **-0.33** | **[-0.49, -0.16]** | **<0.001** | 0.10 | [-0.08, 0.27] | 0.274 |
| Delta age | -0.03 | [-0.11, 0.05] | 0.466 | **-0.14** | **[-0.22, -0.06]** | **<0.001** |
| Average age | -0.01 | [-0.15, 0. 13] | 0.908 | **-0.24** | **[-0.45, -0.04]** | **0.018** |
| Productivity | 0.04 | [-0.11, 0.20] | 0.608 | -0.12 | [-0.26, 0.03] | 0.126 |
| Lay date | **-0.12** | **[-0.18, -0.06]** | **<0.001** | **0.13** | **[0.06, 0.20]** | **<0.001** |
| Delta age x AFR | **0.13** | **[0.08, 0.17]** | **<0.001** | -0.02 | [-0.08, 0.03] | 0.416 |
| Average age x AFR | 0.08 | [0.00, 0.16] | 0.072 | 0.00 | [-0.12, 0.12] | 0.990 |

**Table S2.** Baseline GLMM testing effects of age structure (delta age, average age), sex, productivity, and laying date on nematode burden. Sex interactions with age terms were included to test for sex-specific age trajectories.

| Parameter | *β* | | 95% CI | *p*-value |
| --- | --- | --- | --- | --- |
| *Intercept* | | **2.75** | **[2.58, 2.92]** | **<0.001** |
| Sex (male) | | **0.34** | **[0.15, 0.51]** | **<0.001** |
| Delta age | | **-0.10** | **[-0.16, -0.04]** | **<0.001** |
| Average age | | **-0.15** | **[-0.28, -0.03]** | **0.010** |
| Productivity | | -0.05 | [-0.16, 0.07] | 0.406 |
| Lay date | | 0.00 | [-0.04, 0.05] | 0.940 |
| Sex x Delta age | | 0.00 | [-0.05, 0.06] | 0.978 |
| Sex x Average age | | -0.04 | [-0.22, 0.13] | 0.676 |

**Table S3. Extended GLMM including age at first reproduction (AFR).** Model extending the baseline GLMM by including age at first reproduction (AFR; categorical: age 2, age 3 [reference], and age ≥ 4) as a fixed effect, along with interactions between AFR and age structure terms to test whether recruitment timing modifies age-dependent infection trajectories. All other fixed and random effects are identical to the baseline model.

| Parameter | *β* | 95% CI | *p*-value |
| --- | --- | --- | --- |
| Intercept | **2.69** | **[2.49, 2.88]** | **<0.001** |
| Sex (male) | **0.29** | **[0.10, 0.48]** | **<0.001** |
| Delta age | **-0.09** | **[-0.16, -0.03]** | **0.004** |
| Average age | **-0.14** | **[-0.29, -0.03]** | **0.042** |
| AFR (2) | **0.25** | **[0.04, 0.47]** | **0.016** |
| AFR (3+) | 0.08 | [-0.20, 0.34] | 0.638 |
| Productivity | -0.05 | [-0.15, 0.07] | 0.398 |
| Lay date | 0.00 | [-0.05, 0.05] | 0.974 |
| Sex x Delta age | 0.00 | [-0.05, 0.05] | 0.998 |
| Sex x Average age | -0.01 | [-0.19, 0.17] | 0.882 |

**Table S4.** Summary of GLMMs testing the effects of age at first reproduction (AFR) and age on nematode burdens in male European shags. Coefficients, 95% confidence intervals (CIs) and *p*-values were calculated by parametric bootstrapping (1000 iterations). Significant effects are highlighted in bold.

|  | Delta-age model | | | Chronological-age model | | |
| --- | --- | --- | --- | --- | --- | --- |
| Parameter | *β* | 95% CI | *p*-value | *β* | 95% CI | *p*-value |
| *Intercept* | **3.01** | **[2.80, 3.23]** | **<0.001** | 3.02 | [2.79, 3.24] | **<0.001** |
| AFR (2) | -0.03 | [-0.41, 0.34] | 0.858 | -0.03 | [-0.38, 0.32] | 0.860 |
| AFR (3+) | **-0.94** | **[-1.75, -0.14]** | **0.026** | **-0.91** | **[-1.72, -0.12]** | **0.022** |
| Delta age | **-0.09** | **[-0.19, 0.00]** | **0.054** | – | – | – |
| Average age | 0.02 | [-0.16, 0.17] | 0.826 | 0.17 | [-0.07, 0.41] | 0.150 |
| Chronological age | – | – | – | **-0.19** | **[-0.37, 0.01]** | **0.046** |
| Productivity | 0.05 | [-0.12, 0.19] | 0.586 | 0.05 | [-0.11, 0.22] | 0.566 |
| Lay date | **-0.13** | **[-0.19, -0.06]** | **<0.001** | **-0.13** | **[-0.19, -0.06]** | **0.002** |
| Delta age x AFR (3+) | **0.37** | **[0.22, 0.54]** | **<0.001** | – | – | – |
| Delta age x AFR (2) | -0.06 | [-0.14, 0.03] | 0.182 | – | – | – |
| Average age x AFR (3+) | 0.27 | [-0.16, 0.69] | 0.200 | -0.36 | [-0.87, 0.15] | 0.174 |
| Average age x AFR (2) | **-0.49** | **[-0.89, -0.07]** | **0.024** | -0.38 | [-0.82, 0.03] | 0.062 |
| Chronological age x AFR (3+) | – | – | – | **0.72** | **[0.40, 1.06]** | **<0.001** |
| Chronological age x AFR (2) | – | – | – | -0.11 | [-0.29, 0.06] | 0.156 |
|  |  |  |  |  |  |  |
| *Random* |  | *Variance (SD)* |  |  | *Variance (SD)* |  |
| Bird ID (intercept) |  | 0.115 (0.340) |  |  | 0.115 (0.340) |  |
| Year (intercept) |  | 0.049 (0.210) |  |  | 0.049 (0.210) |  |
| R^2^ marginal |  | 0.354 |  |  | 0.354 |  |
| R^2^ conditional |  | 0.870 |  |  | 0.870 |  |

**Table S5.** Summary of GLMMs testing the effects of age at first reproduction (AFR) and age on nematode burdens in female European shags. Interactions between AFR and delta-age, and between AFR and average-age, were dropped from models when non-significant (see Methods). Coefficients, 95% confidence intervals (CIs) and *p*-values were calculated by parametric bootstrapping (1000 iterations). Significant effects are highlighted in bold.

|  | Delta-age model | | | Chronological-age model  (selective disappearance) | | |
| --- | --- | --- | --- | --- | --- | --- |
| Parameter | *β* | 95% CI | *p*-value | *β* | 95% CI | *p*-value |
| *Intercept* | **2.73** | **[2.48, 2.96]** | **<0.001** | **2.73** | **[2.50, 2.97]** | **<0.001** |
| AFR (2) | -0.01 | [-0.42, 0.40] | 0.932 | -0.02 | [-0.42, 0.38] | 0.93 |
| AFR (3+) | 0.10 | [-0.23, 0.43] | 0.558 | 0.09 | [-0.27, 0.45] | 0.620 |
| Delta age | **-0.15** | **[-0.23, -0.06]** | **0.002** | – | – |  |
| Average age | **-0.21** | **[-0.38, -0.05]** | **0.008** | 0.03 | [-0.19, 0.26] | 0.730 |
| Productivity | -0.13 | [-0.27, 0.02] | 0.080 | -0.12 | [-0.27, 0.03] | 0.106 |
| Lay date | **0.13** | **[0.07, 0.20]** | **<0.001** | **0.13** | **[0.06, 0.19]** | **<0.001** |
|  |  |  |  |  |  |  |
| *Random* |  | *Variance (SD)* |  |  | *Variance (SD)* |  |
| Bird ID (intercept) |  | 0.232 (0.482) |  |  | 0.232 (0.482) |  |
| Year (intercept) |  | 0.043 (0.208) |  |  | 0.043 (0.208) |  |
| R^2^ marginal |  | 0.203 |  |  | 0. 203 |  |
| R^2^ conditional |  | 0.859 |  |  | 0.859 |  |
